# Structural Model Analysis of Multiple Quantitative Traits

**DOI:** 10.1371/journal.pgen.0020114

**Published:** 2006-07-21

**Authors:** Renhua Li, Shirng-Wern Tsaih, Keith Shockley, Ioannis M Stylianou, Jon Wergedal, Beverly Paigen, Gary A Churchill

**Affiliations:** 1 The Jackson Laboratory, Bar Harbor, Maine, United States of America; 2 Musculoskeletal Disease Center, J. L. Pettis Memorial VA Medical Center, Loma Linda University, Loma Linda, California, United States of America; 3 Department of Medicine, Loma Linda University, Loma Linda, California, United States of America; 4 Department of Biochemistry, Loma Linda University, Loma Linda, California, United States of America; University of Alabama at Birmingham, United States of America

## Abstract

We introduce a method for the analysis of multilocus, multitrait genetic data that provides an intuitive and precise characterization of genetic architecture. We show that it is possible to infer the magnitude and direction of causal relationships among multiple correlated phenotypes and illustrate the technique using body composition and bone density data from mouse intercross populations. Using these techniques we are able to distinguish genetic loci that affect adiposity from those that affect overall body size and thus reveal a shortcoming of standardized measures such as body mass index that are widely used in obesity research. The identification of causal networks sheds light on the nature of genetic heterogeneity and pleiotropy in complex genetic systems.

## Synopsis

Disease states are often associated with multiple, correlated traits that may result from shared genetic and nongenetic factors. Genetic analysis of multiple traits can reveal a network of effects in which each trait is influenced by more than one genetic locus (heterogeneity) and different traits share one or more loci in common (pleiotropy). Physiological interactions independent of genetic factors may also contribute to the observed correlations. Structural equation modeling is proposed as a statistical method to characterize the architecture of multiple trait genetic systems. Application of structural equation modeling to body size, adiposity, and bone geometry traits illustrates how the effects of a genetic locus can be decomposed along direct and indirect paths that may be mediated through interactions with other traits. Using this technique the authors identify adiposity loci that act independently of loci affecting overall body size.

## Introduction

The most common and pervasive human health problems, including heart disease, osteoporosis, diabetes, and cancer, result from the complex interaction of multiple genetic and environmental factors. Disease states are often associated with multiple, correlated traits, referred to as subphenotypes. The ability to assay subphenotypes of a disease state presents a unique opportunity to investigate the mechanisms underlying disease susceptibility and progression [1]. Observed associations among these traits may be driven by common genetic factors or may result from physiological interactions [1,2]. For example, low- and high-density lipoprotein cholesterol, triglycerides, blood pressure, plasma insulin levels, and C-reactive proteins are all measurable phenotypes associated with cardiovascular disease. We would like to know whether these phenotypes share common genetic determinants, which genetic factors are specific to different subphenotypes, and the nature of the nongenetic interactions among these phenotypes.

The genetic analysis of complex traits is facilitated by the study of inbred line crosses using animal models, typically rodents. The genetically varied progeny from a cross can be reared in a controlled environment, and multiple quantitative phenotypes that are relevant to a disease outcome can be measured in individual animals [3]. Genetically randomized experimental populations that segregate naturally occurring allelic variants can provide a basis for the inference of networks of causal associations among genetic loci, physiological phenotypes, and disease states. The inbred cross experimental design provides a setting in which the direction of causality, from genes to phenotypes, can be inferred unambiguously. The randomization of genetic variants that occurs during meiosis provides a setting that is analogous to a randomized experimental design and thus admits causal inferences [4], consistent with our intuition that variation in genetic factors causes phenotypic variation.

Multivariate analysis of quantitative traits can be used to investigate the structure of a genetic system that includes allelic variation at multiple loci, intermediate phenotypes, and disease states [5]. Jiang and Zeng [6] proposed a method for quantitative trait locus (QTL) detection based on a multivariate normal model with unconstrained covariance structure. Alternatively, dimension reduction techniques, such as principal component analysis, can be applied to sets of correlated traits [7]. Multivariate QTL analyses can provide enhanced power and resolution in QTL mapping when traits are highly correlated and share common genetic determinants [8]. However, neither QTL analysis nor dimension reduction techniques provide insight into the relationships among the phenotypes or the differential effects of the genetic loci. Mapping studies that investigate clusters of related phenotypes often reveal a network of genetic effects, in which each phenotype is influenced by multiple loci (heterogeneity) and different phenotypes share one or more loci in common (pleiotropy) [1,5]. The complexity of observed QTL networks will vary depending on the traits and the power of the study design. It is also likely that physiological interactions independent of genetic factors may result in correlated phenotypic responses [2]. For example, heart rate and blood pressure will covary when measured simultaneously on the same animal. The methods described here represent a next step in the analysis of the genetic architecture of multiple traits and QTLs that have been detected using either univariate or multivariate genome scans.

Structural equation models (SEMs), also known as path models [9], are related to Bayesian networks [10–12]. In each of these approaches, we can represent the model structure as a directed graph in which measured variables are represented as nodes and causal relationships are represented as directed edges between the nodes. Multivariate probability distributions are defined by the conditional dependencies among variables represented in the graphical model [12]. Bayesian networks emphasize probabilistic relationships among discrete variables, whereas SEMs emphasize the correlation structure of continuously variable data. SEMs are an extension of standard multiple regression techniques. They impose structure on the expected correlation through a system of linear equations that define the causal relationships among measured variables in a system. Covariates—factors such as sex, batch, or litter that are external to direct genetic causality but introduce variations in phenotypes of interest—can be incorporated into SEM analysis [13]. SEM is essentially a hierarchical system of regression relationships in which any given variable may be both a response and a predictor.

Herein, we propose a SEM approach to analyze complex genetic systems using mouse inbred crosses. SEM methodology has been applied in several studies of human inheritance with the aim of improving QTL detection [14–19]. The approach presented here does not explicitly use structural modeling for detection. Instead, we focus on SEM as a descriptive and inferential tool to investigate the simultaneous effects of QTLs on multiple phenotypes and interactions among those phenotypes. The relationships among QTLs and phenotypes can be tested and quantified to establish the nature of genetic heterogeneity, pleiotropy, and the role of physiological pathways in mediating genetic effects [20,21]. We illustrate the approach with an analysis of two phenotypes related to obesity: one, an SM/J × NZB/B1NJ intercross [22], and two, bone phenotypes in a NZB/B1NJ × RF/J intercross [23]. For brevity, these strains will be denoted SM, NZB, and RF.

## Materials and Methods

### Mouse Intercross Populations

The SM × NZB intercross population of 260 female and 253 male mice was raised on an atherogenic diet for 16 wk starting at 8 wk of age. At 24 wk we obtained total body weight and weights of the inguinal, gonadal, peritoneal, and mesenteric fat pads. Lean body weight was computed by subtracting the total of the fat pad weights from the body weight. In the analyses described here, all traits were square-root transformed to obtain the best linear relationships. Additional information on husbandry, phenotyping, and genotyping of this cross can be found in Stylianou et al. [22].

The NZB × RF intercross population consists of 661 female mice raised on standard (4% fat) diet. At 10 wk of age, femurs were isolated and their geometric properties were determined by peripheral quantitative computed tomography. We consider body weight and two bone geometry traits, femur length and the periosteal circumference of the femur (PCIR). All traits were log transformed to obtain the best linear relationships. Additional information on this cross can be found in Wergedal et al. [23].

The Institutional Animal Care and Use Committee of The Jackson Laboratory approved all experimental protocols. All data used in this study are available at http://www.jax.org/staff/churchill/labsite/datasets/qtl/qtlarchive.

### Structural Equation Models

In structural equation modeling, variables are standardized by centering on sample means. Thus the variances and covariances are the parameters of interest. The key idea behind SEM is that causal relationships among the variables determine the expected pattern of correlations [24]. A SEM represents causal relationships among measured and latent variables both graphically and as a set of linear equations, the *structural equations*, that define the interactions among the variables. In the graphical representation of a SEM, a variable with an arrow pointing to it is termed an *endogenous* variable, which is similar to a *response* or *dependent* variable in regression terminology. An endogenous variable is causally affected by the state of at least one other variable in the model. A variable without an arrow pointing to it is termed an *exogenous* variable, which is similar to an *independent* variable or *predictor* in regression terminology. Exogenous variables are upstream of all causal effects in the model. Variables that are connected through a single edge in the graphical model are said to have *direct path*. An effect that is mediated through other measured variables is represented by an *indirect path* with more than one edge. Variables may be connected by more than one path. The sign and magnitude of the direct effects in the graphical model are represented by *path coefficients* in the structural equations.

The endogenous variables in a SEM are assumed to follow a multivariate normal distribution, while exogenous variables can be either continuous or categorical. The maximized log likelihood function takes the form [25,26]:

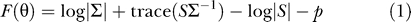
where *θ* is a vector of model parameters that includes path coefficients, variances of all exogenous variables, and covariances for all pairs of exogenous variables; *p* is the number of variables included in the model; Σ is the predicted covariance matrix and is implicitly a function of the model parameters; *S* is the observed covariance matrix; and |*X*| denotes the determinant of a matrix *X*. The number of unique elements in a covariance matrix is *p*(*p* + 1)/2 due to symmetry, and the number of model parameters is denoted by *q*. Intuitively, the parameter values are chosen to give a predicted covariance matrix that is as similar as possible to the observed covariance matrix, subject to the constraints imposed by the structural equations. A critical quantity in determining our ability to fit and assess a model is the residual degrees of freedom (df), where df = *p*(*p* + 1)/2 − *q*. If the residual df are less than 1, the model has as many or more parameters than data points and thus the goodness-of-fit cannot be assessed.

### Causal Inference

**Figure 1:**
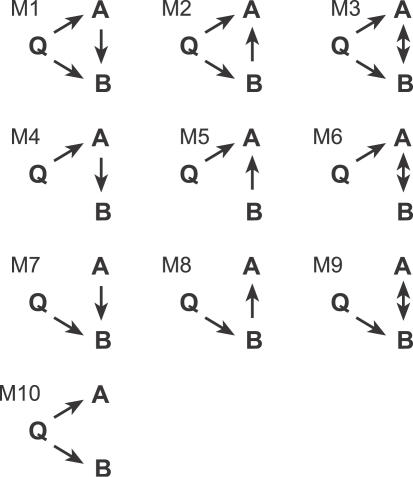
Causal Relationships among a QTL and Two Phenotypes Single-headed arrows indicate causal effects and doubled-headed arrows indicate unresolved associations between the two phenotypes. Phenotypes are indicated by A and B; QTL by Q.

To illustrate causal inference in the context of QTL mapping, consider the possible relationships between a genetic locus, Q, and two correlated traits, A and B, as in Figure 1. In each of the models M1–M9, there is a direct causal connection between A and B that represents a physiological interaction. In model M10 the correlation between A and B is indirect—a result of the shared QTL. Directed paths represent causal effects, and bidirectional paths represent undirected associations. The latter may indicate the presence of an unobserved factor that influences both traits. The QTL may be pleiotropic, with direct effects on both traits, as in models M1–M3 and M10. Alternatively, the QTL may have a direct effect on only one trait.

Causal effects from a QTL to a phenotype have a defined direction. The causal inference follows as a consequence of meiotic randomization of genetic factors in the inbred line cross. The causal inference can be extended to include the relationships among phenotypes by considering both the unadjusted and partial correlation relationships among the variables. For example, in model M7, Q and A are unconditionally independent. However, conditioning on B will result in a nonzero partial correlation. By contrast, in M8, Q and A will be unconditionally correlated. Conditioning on B breaks the causal chain from Q to A, and their partial correlation will be zero. When all three variables are causally connected, as in models M1–M3, the raw and partial correlations will all be nonzero, but they will change in magnitude depending on the signs of the path coefficients. The relationships among the various raw and partial correlations are subject to statistical fluctuations but they can be captured in the log of odds ratio (LOD) scores, as described below, and these form the basis for constructing multilocus SEMs.

### Development of a Structural Equation Model

The process of developing a SEM for genetic mapping data is described in five steps, below. Steps 1 and 2 involve QTL detection and they employ standard QTL detection methods and serve the purpose of identifying the variables that will be used in the structural modeling. Once the QTL have been identified, their genotypes are imputed [27] and the covariance matrix of all traits and QTLs are computed by averaging over imputations. This estimated covariance matrix is analyzed in steps 3 through 5 to build, assess, and revise the SEM. Some iteration among these steps may be required to arrive at a suitable model. However, extensive model refinement may lead to a model that fits the existing data but does not generalize well (i.e., overfitting) and is not recommended.

#### Step 1. Identify QTLs for individual phenotypes.

We use genome scans to identify the genetic loci that will be included in the SEM [27]. A single locus genome scan is based on the linear model

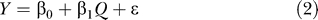
where *Y* is a vector of trait values, *β*_0_ is the population mean, *Q* is a vector of QTL genotypes, *β*_1_ is the QTL effect, and ɛ is the residual vector. The location of the QTL is scanned over the entire genome and a LOD score (or equivalently, likelihood ratio) is used to determine if a QTL is present. If covariates, such as sex, are important predictors of the trait values, these should be included in the linear model underlying the genome scans [13]. In addition, the traits may be scanned using a pairwise genome scan to identify epistatic QTLs [27].

#### Step 2. Identify pleiotropic QTLs.

In this step we perform conditional genome scans using one trait as a covariate in the analysis of another trait. The choice of which trait(s) to use as covariates may be dictated by the known biological relationships among the traits, or it may be carried out systematically. In this setting, factors that are believed to be upstream in causal pathways should be employed as conditioning variables. The linear model used in the conditional genome scans is

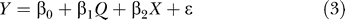
where *X* is the conditioning variable and *β*_2_ is the effect of *X* on the response *Y,* adjusted for the *Q* effect.

Comparison of the unconditioned and conditioned scans may reveal a substantial change in the LOD score at a locus. If the change (ΔLOD) is large in absolute value, this suggests that the variable *X* is causally connected to *Q* and to *Y*. We use a critical value of 2.0 as a guideline, corresponding to a 0.05 type I error rate based on simulations in which the conditioning variable is unrelated to the response. The significance of these edges will be evaluated further in subsequent steps.

The direction of change in the LOD score upon adjustment for a covariate depends on the signs of QTL effects on the trait and on the covariate, as well as the sign of the path coefficient connecting the trait and the covariate. When both the direct and indirect paths share the same sign, the LOD score in the conditional scan will be smaller than the unconditional LOD score. When the direct path and the indirect path have opposite effects, the conditional LOD score will be greater than the unconditional LOD score. In this case, the direct and indirect effects cancel one another and the QTL effect is reduced in the unconditional scan. It follows that changes in the LOD upon conditioning can be used to infer the sign of QTL effects along different paths.

#### Step 3. Define an initial path model.

In the graphical SEM, each measured trait is represented as a node; QTLs identified in steps 1 and 2 are also included as nodes. Edges should be directed from the QTL nodes to the corresponding traits. When a significant ΔLOD value is observed, edges should be directed from the QTL to each of the traits, and an edge from the conditioning trait to the response should also be added. The significance and causal direction of these edges should be examined in model refinement (step 5).

In many instances, a SEM that includes only measured variables will be sufficient. However, latent variables [28] representing unmeasured or hypothetical quantities that can be inferred from other measured variables, may be incorporated in a SEM. Addition of a latent variable can effectively reduce the dimensionality of the data when several highly correlated variables are being influenced by an underlying quantity that is not directly observed.

##### Step 4. Assessment of the model.

This step involves a comparison of the predicted and observed covariance matrices, t-tests for individual path coefficients, and consideration of other model diagnostics. The goodness-of-fit test statistic is (*N* − 1)*F(θ),* where *N* is the sample size and *F(θ)* is defined in Equation 1. It follows a χ^2^ distribution with df *p*(*p* + 1)/2 − *q*. Significant values (*p* < 0.05) indicate that the model does not provide a good fit to the data. Each path coefficient should be individually significant (*p* < 0.05) using a t-test (Wald's test). The maximum standardized residual difference between the observed and predicted covariance matrices should be small. The root mean square error of approximation measures the lack of fit of the model to the theoretic population covariance matrix and values of 0.05 or less indicate an acceptable fit. A model that does not provide an accurate prediction of the observed covariance should be refined.

##### Step 5. Refine the model.

Model refinement involves the proposal and assessment of a new model. The procedures used to suggest a new model include adding a new path to the initial model, removing a path, or reversing the causal direction of a path [9]. When a new path is added to an existing model it should result in a significant change in the goodness of fit statistic (> 3.84 for 1 df). This is the likelihood ratio test. When an existing path is removed from the model, the change in the goodness of fit statistic should be nonsignificant. Particular attention should be paid to the edges between phenotypes. Whereas the direction of causality from QTL to a trait is clear, the direction of causal connections between traits should be carefully examined by exploring all possible alternatives, as illustrated below.

Additionally, one may consider model selection methods. For example, Akaike's information criterion (AIC) [29], provides a penalized likelihood statistic for model comparison. CAIC is an adjustment of AIC to correct for bias in small samples and is recommended when the ratio of the sample size to the number of estimable parameters is less than 40 [30]. In our examples this ratio is around 10. A model with the smallest value of AIC or CAIC among several candidate models is preferred. The expected value of the cross-validation index (ECVI) estimates the overall error and predictive validity of a model [31]. ECVI values may be compared among several models. An interval estimate is helpful to avoid paying too much attention to small differences.

Model refinement and assessment (steps 4 and 5) are often carried out iteratively. A final model should meet several standards [9]: (1) it should be identified or overidentified with at least 1 residual df; (2) the *p*-value associated with the goodness-of-fit test should be greater than 0.05; (3) the largest standardized residual should not exceed 2.0 in absolute value; (4) individual path coefficients should be significantly different from zero based on the t-test; (5) standardized path coefficients should not be trivial (absolute values exceed 0.05); and (6) a substantial proportion of phenotypic variance of the endogenous variables should be explained by the model. If all of these standards are met, we may conclude that the model provides a reasonable description of the data.

### Modeling Genetic Effects

In the context of an intercross (F2) population, each QTL has three possible genotypic states. We can assume intralocus additivity by encoding genotypes as 0, 1, or 2, and treating these scores as a continuous variable in the SEM. Alternatively, the genetic effect is represented as a pair of 0,1 (dummy) variables. The most widely used parameterization, following Cockerham [32], partitions the genetic effects into additive (A) and dominant (D) components. The A and D components are orthogonal to one another and thus we can fix their covariances in the SEM to be zero. In the model-fitting and refinement steps, we always retain or drop both components as a unit, even if only one component is significant. Epistatic interactions for pairs of QTLs can be partitioned into four components [32], additive × additive (AA), additive × dominant (AD), dominant × additive (DA) and dominant × dominant (DD). These are also orthogonal and are treated as a single unit in the model. If an epistatic term is included in the model, we retain the main effect terms, regardless of their marginal significance, to ensure that the model is interpretable. Although we considered epistatic terms in the examples below, we found that the added explanatory power was not sufficient to justify the additional free parameters in the model. With larger sample sizes it may become practical to include epistatic effects in a SEM.

### Path Analysis

One important feature of SEM is that direct and indirect effects of a QTL on a trait can be distinguished, and the relative strengths of effects along different paths can be calculated and compared [20,21]. The effect of a direct path from a QTL to a trait is represented by the path coefficient. Path coefficients are typically standardized relative to the residual variance of the endogenous variable to which they are directed. Thus all error terms in a standardized model have variance 1, and path coefficients are expressed in standard deviation units. In this way all path coefficients are directly comparable and are independent of the original measurement scale. The effect of an indirect path is the product of all the standardized path coefficients (including + and − signs) along this path. The total effect of the QTL on a trait is the sum of the effects along all the direct and indirect paths connecting the two variables.

We note that the sign of a path coefficient from a QTL to a trait is determined by the choice of the reference genotype (encoded as zero). If the effect of an allelic substitution away from the reference is to increase the mean trait value, the sign of the path coefficient is positive. If allelic substitution away from the reference causes the trait value to decrease, the sign is negative. In the examples below, the homozygous NZB genotype is our reference. The same interpretation applies to path coefficients for categorical covariates such as sex. In the models below we have used female as the reference.

### Computing

Genome scans were carried out in the MATLAB software environment (MathWorks, Natick, Massachusetts, United States) using the pseudomarker package (v2.02) [27]. Scans were conducted at 2 cM resolution using the imputation method with 256 imputations. Significance of QTLs was assessed using permutation analysis [33] with 1,000 permutations. Genome-wide significance is defined as the 95th percentile of the maximum LOD score and suggestive QTLs exceed the 37th percentile. Structural equation modeling analysis was carried out using the PROC CALIS procedure in the SAS software package (SAS, Cary, North Carolina, United States) or in AMOS 6.0 (SPSS, Chicago, Illinois, United States). QTLs were represented by the marker nearest to the LOD peak, and missing genotypes (< 5%) were inferred from flanking marker data.

## Results

### Measuring Adiposity

We first consider the problem of defining a measure of adiposity. Fat pad weights and lean body weight are positively correlated in the SM × NZB cross population. Fat pad weight by itself is inadequate as a measure of obesity. Two animals with similar fat pad weights may have different body size such that one animal is considered obese and the other not. Thus it is important to consider the fat pad weight relative to the size of the animal. In our analysis we used the lean body weight as surrogate for size. The lean body weight is computed by subtracting the weights of the four major fat pads from the total body weight and thus will include a small contribution from other adipose tissues.

**Figure 2:**
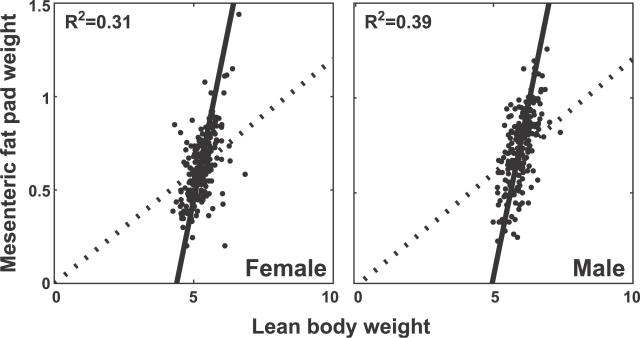
Relationship of Mesenteric Fat Pad Weight to Lean Body Weight for Female and Male Animals Both phenotypes are square root transformed. The dotted line indicates the ratio standard of constant adiposity index. The solid line is the regression of mesenteric fat pad weight on lean body weight.

There are two common approaches to defining a relative measure. First, one could compute an *adiposity index* as the ratio of fat pad weight to lean body weight. Second, one could regress fat pad weight on lean body weight and consider the deviation from the regression line (the residual) as a measure of adiposity. Although the regression approach is clearly preferred, ratio standardization is still widely used [34,35]. To see why this is so, consider the implications of using each measure. The ratio standard assumes a proportional relationship *(y* = *bx)* between the variables, which implies that the regression line relating these traits should pass through the origin. One might argue that a hypothetical animal with zero lean body mass should also have a fat pad mass of zero. However, the argument is fallacious, as it extrapolates beyond the range of the data over which the linear approximation is valid. Regression standardization allows one additional df in the form of an intercept term *(y* = *a* + *bx)*. The relationship of mesenteric fat pad weight to lean body weight is shown graphically in Figure 2. The regression line and the ratio line meet at the mean of each trait but the two diverge away from the mean point. The regression line fits the data well over its entire range. For either standard, adiposity is measured as the deviation from the fitted line. In this case, the ratio standard is severely biased. An animal with higher than average lean body weight and normal adiposity according to the regression standard would be considered obese by the ratio standard. The converse would be true for an animal with lower than average lean body weight. Therefore we conclude that the regression method provides a more appropriate adjustment of fat pad weight for lean body weight and is to be preferred as a measure of adiposity. SEMs generalize the regression model and thus provide a regression standardization.

### Body Size and Fat Pad QTL

**Figure 3:**
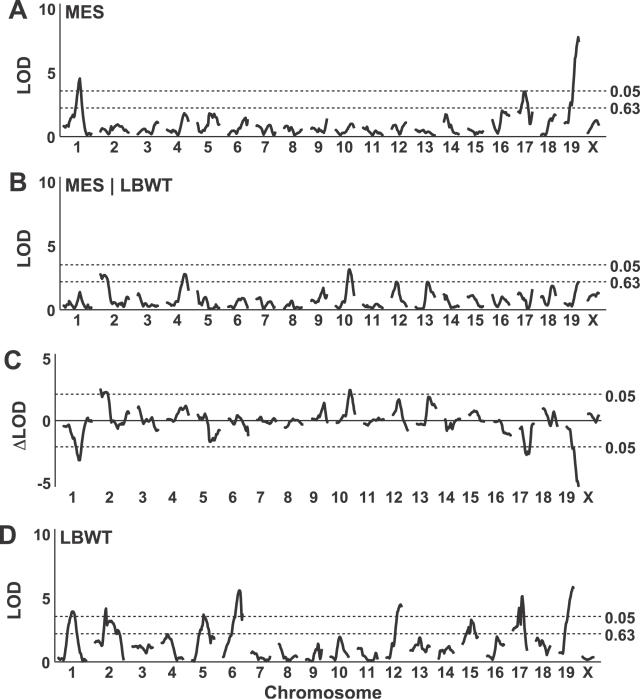
Genome-Wide Scans for Mesenteric Fat Pad Weight at 2 cM Resolution Genome scans shown are (A) mesenteric fat pad weight with sex as an additive covariate; (B) mesenteric fat pad weight with sex and lean body weight as additive covariates; (C) difference in LOD scores between scans in (A) and (B); and (D) lean body weight with sex as an additive covariate. LBWT, lean body weight; MES, mesenteric fat pad weight.

A genome scan of the mesenteric fat pad weight (with sex as an additive covariate) reveals significant QTLs on Chromosomes 1, 17, and 19 (Figure 3A). A second genome scan for mesenteric fat pad weight, using both sex and lean body weight as additive covariates, shows suggestive QTL peaks on Chromosomes 2, 4, and 10 (Figure 3B). The differences between these two scans are dramatic, with both upward and downward changes in the LOD scores (Figure 3C). The peak LOD score decreases after adjustment on Chromosomes 1, 17, and 19, and it increases after adjustment on Chromosomes 2 and 10. A genome scan of the lean body weight (adjusted for sex) reveals QTLs on Chromosomes 1, 2, 5, 6, 12, 15, 17, and 19 (Figure 3D). The substantial overlap with QTLs for fat pad weight suggests that pleiotropic effects are an important component of the genetic architecture. One goal of structural equation modeling is to translate the observed changes in LOD scores following adjustment for a covariate into an interpretation of the nature of these pleiotropic effects.

We note here that a genome scan of the adiposity index (unpublished data) reveals only two significant QTLs, one on Chromosome 19 and the other on Chromosome 1, and there are no suggestive QTLs. As indicated below, these two loci primarily affect the overall body size and are not specifically impacting adiposity. Genome scans for all four fat pad traits are provided in Figure S1.

### A SEM of Mesenteric Fat Pad Weight

**Figure 4:**
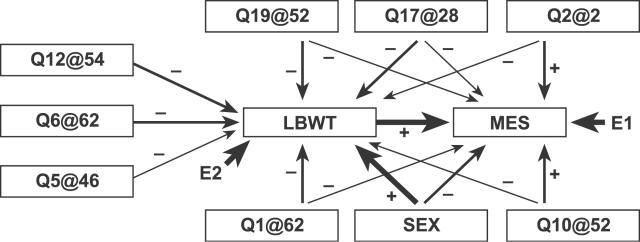
Graphical Representation of the SEM for Mesenteric Fat Pad Weight Genetic loci are indicated by Q followed by the chromosome number, and @ followed by the cM position of the LOD peak. Single-headed arrows indicate causal paths, and the thickness of each arrow is proportion to the effect size (path coefficient). A negative sign from a QTL to a trait indicates that the NZB allele is associated with high trait values. E1 and E2 denote unobserved residual error.

As a starting point for the development of a SEM we consider all of the QTLs that are significant (*p* < 0.05, genome-wide adjusted) in at least one of the genome scans and any suggestive QTLs that have significant ΔLOD values. Directed edges from each QTL to the corresponding trait are included in the graphical SEM. QTLs with a ΔLOD exceeding 2 in absolute value are connected with directed edge to both traits. In addition, a directed edge is tentatively connected from lean body weight to the fat pad weight. We used an additive model of the genetic effects. This initial model is assessed and refined, using the steps described in Materials and Methods, to arrive at the model shown in Figure 4. In this case, no refinements were made to the initial model. The path coefficients are all significantly different from zero (Table 1), the proportion of variance explained by the model is 46.5% for mesenteric fat pad mass and 54.4% for lean body weight, and the maximized standard residual is 0.93. Several goodness-of-fit statistics for model M1 are shown in Table 2. These measures all indicate that fit of the model is acceptable.

**Table 1:**
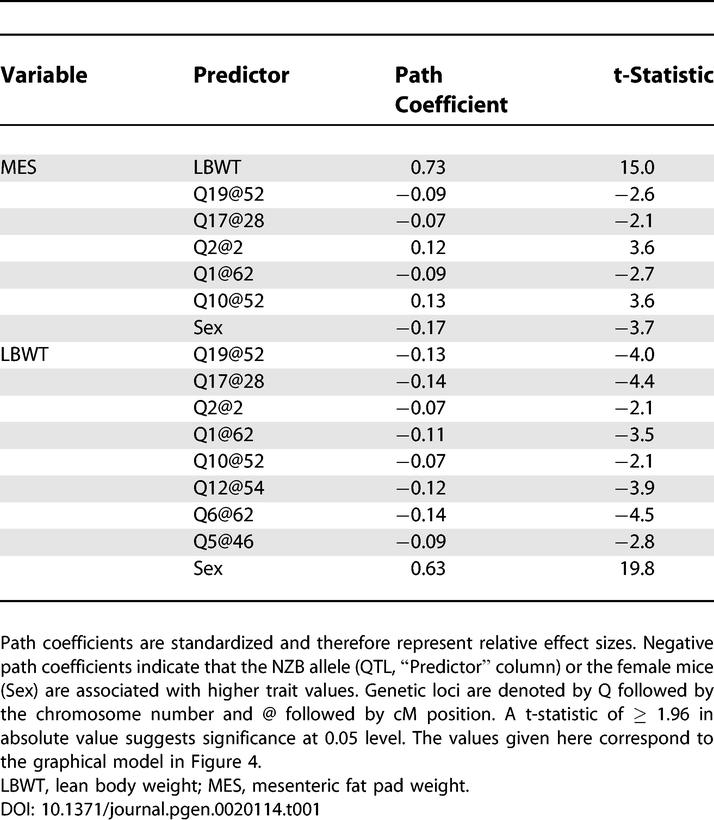
Structural Equations of the Mesenteric Fat Pad Model

**Table 2:**
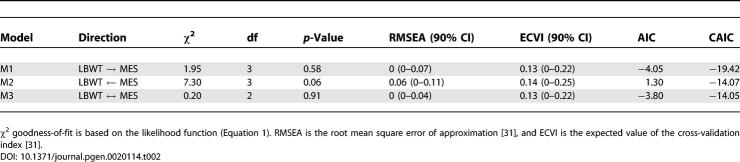
Model Comparison for Mesenteric Fat Pad Mass and Lean Body Weight

In order to assess the direction of causality between lean body weight and mesenteric fat pad weight, we consider the models listed in Table 2. These models are derived from the initial model by varying the direction of the causal relationship between the traits. In general it may be necessary to allow for model refinement but none was required here. Goodness-of-fit statistics indicate that the model, M2, in which mesenteric fat weight is upstream does not fit. The overall fit of the bidirectional model, M3, is acceptable, but a t-test (*t* = 1.48) indicates that the path coefficient from mesenteric fat pad weight to lean body weight is nonsignificant. M1 also has the smallest CAIC value (Table 2) and is preferred over the other models. We conclude that lean body weight is causal to fat pad weight. This is consistent with our intuition that a mouse with a larger body size will tend to have larger fat pads. Caution is recommended in this interpretation, because both traits represent a composite of multiple causal effects, some of which are mediated by unobserved factors. The SEM indicates that the net effect is unidirectional.

A unique feature of SEMs is the ability to resolve the sign and magnitude of effects along multiple direct and indirect paths. Path analysis provides a detailed description of the pleiotropic effects in the system that takes into account multiple genetic and nongenetic sources of variation. For those QTLs that are associated with both mesenteric fat pad weight and lean body weight, path analysis establishes the relative contributions of the direct and indirect effects. Decomposition of QTL effects along direct and indirect paths is illustrated in Table 3, using two QTLs as examples (see Tables S1–S4 for a complete set). The locus Q19@52 shows negative effects on mesenteric fat pad weight along both the direct and indirect paths, indicating that this locus affects mesenteric fat pad weight in the same direction through two physiological pathways. The peak LOD score in an unadjusted genome scan is a reflection of the net effect over all paths. The contribution from an indirect path can be blocked by conditioning on the intermediate variable. In this case, the result is a downward change in LOD score in the conditional scan. In contrast, the Q10@52 shows opposite effects on mesenteric fat pad weight along direct and indirect paths. Conditioning on lean body weight blocks the indirect path, resulting in an increased LOD score. In this way, changes in LOD score upon conditioning provide information about the signs of path coefficients in the SEM.

**Table 3:**
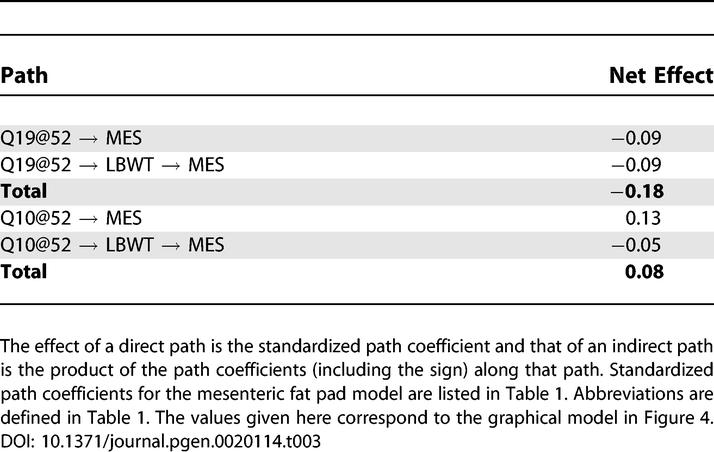
Path Analysis of QTL Effects

In the SEM for mesenteric fat pad weight (Figure 4 and Table 1) we can identify three distinct classes of QTL effects. The QTLs on Chromosomes 5, 6, and 12 have direct effects on lean body weight and only indirect effects on the fat pad weight. The QTLs on Chromosomes 1, 17, and 19 have pleiotropic “body size” effects. NZB alleles at these loci are associated with increased lean body weight and increased fat pad weight. The QTLs on Chromosomes 2 and 10 may be interpreted as “adiposity” loci. At these loci, SM alleles are associated with smaller lean body weight and larger fat pad weights. The largest influence on mesenteric fat pad weight is through its positive correlation with lean body weight. Females have relatively larger fat pad weights. The largest genetics effects are on Chromosomes 2 and 10, where SM alleles contribute to increased adiposity. The largest single effect on lean body weight is sex, but the total effect of multiple genetic loci (with all high alleles contributed by NZB) is greater still.

### Modeling Adiposity and Body Weight

**Figure 5:**
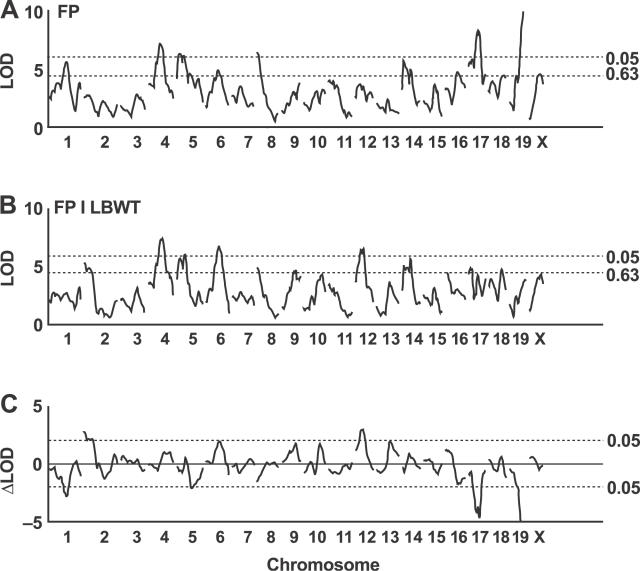
Genome-Wide Scans for Multiple Fat Pad Traits at 2 cM Resolution Genome scans shown are (A) the four fat pad traits (inguinal, gonadal, peritoneal, and mesenteric fat pad weight) with sex as an additive covariate; (B) the four fat pad traits with sex and lean body weight as additive covariates; and (C) the difference in LOD scores between scans in (A) and (B). FP, the four fat pad traits; LBWT, lean body weight.

Univariate and multivariate genome scans [6] were performed for the four fat pad traits. The multivariate scan (Figure 5A) detected all of the QTLs that were detected by scanning each fat pad trait one at a time (Figure S1), and a new QTL was detected on Chromosome 14. The QTLs on Chromosomes 1, 2, 5, 6, 12, 17, and 19 show significant changes in LOD scores (ΔLOD > 2), when conditioning on lean body weight (Figure 5C). In our initial SEM, we included all QTLs that were significant in any of the genome scans and all suggestive QTLs that showed a significant (> 2) change in LOD score between these two multivariate genome scans.

**Figure 6:**
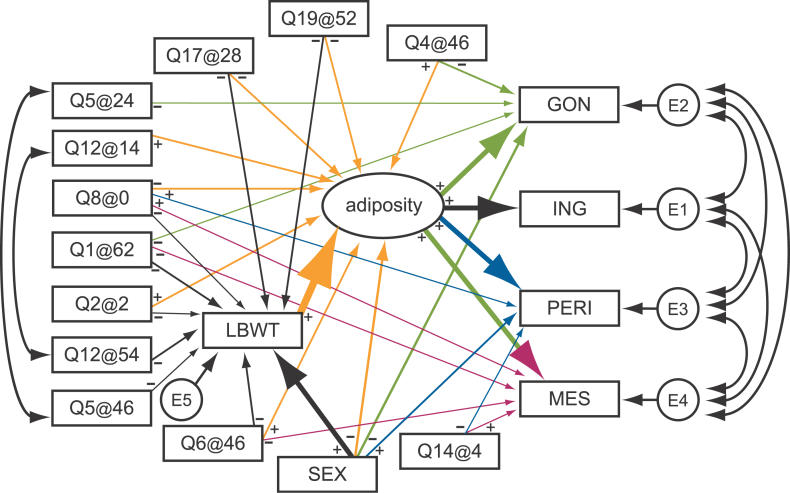
Structural Equation Model for Adiposity and Lean Body weight The four fat pad traits are gonadal (GON); inguinal (ING); mesenteric (MES); and peritoneal (PERI). Single-headed arrows indicate causal paths, and the thickness of each arrow is proportional to the effect sizes. Doubled-headed arrows denote unresolved covariance. The boxes indicate measured traits or QTL and the oval denotes a latent variable. E1, E2, E3, E4, and E5 denote unobserved residual error. The negative sign from a QTL to a trait indicates that the NZB allele is associated with high trait values.

Correlations among the fat pad weights were all about 0.7, thus multicollinearity is a concern for developing a SEM. To address this, we introduced a latent variable to capture the shared variation among the fat pad weights and refer to this latent variable as “adiposity.” Variance in any of the fat pad traits that is unrelated to adiposity is captured by the residual error variances. We formulated an initial model using only main effect terms and additive genetic effects as described in Materials and Methods. The population size of 531 limits the size of the models that we can consider. A final model, obtained after a few iterations of model refinement, is shown in Figure 6. The model selection and goodness of fit statistics are summarized in Table 4.

**Table 4:**
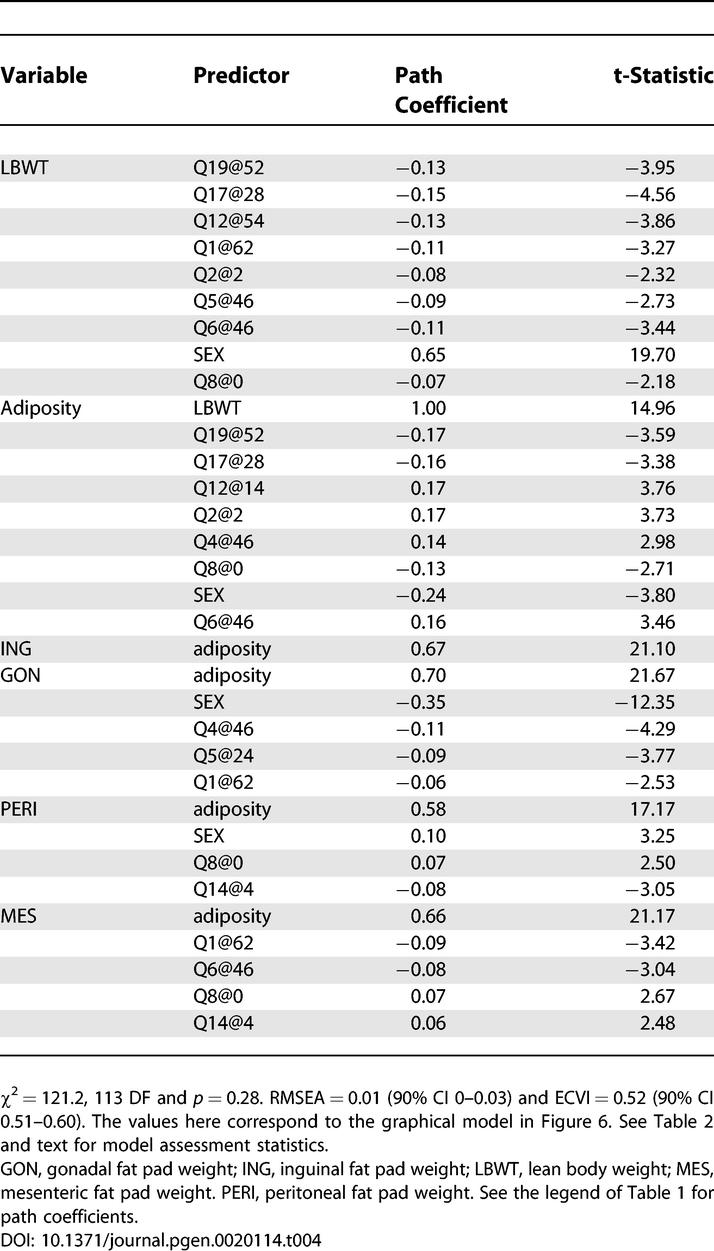
Model Assessment and Path Coefficients for the Adiposity SEM

Several conclusions are available from inspection of the graphical model in Figure 6. For example, we see that female mice have lower body weight, higher adiposity, larger gonadal fat pads, and smaller peritoneal fat pads compared to males. SM alleles on Chromosomes 17 and 19 are associated with lower adiposity and lower body weight compared to NZB alleles. The Chromosome 2 QTL is associated with higher adiposity and lower lean body weight. QTLs specific to adiposity or fat pad traits include Q4@46, Q5@24, Q12@14, and Q14@4. QTLs specific to lean body weight include Q5@46 and Q12@54. Each QTL contributes to overall body composition with a unique pattern of effects.

### Modeling Bone Geometry and Body Weight

**Figure 7:**
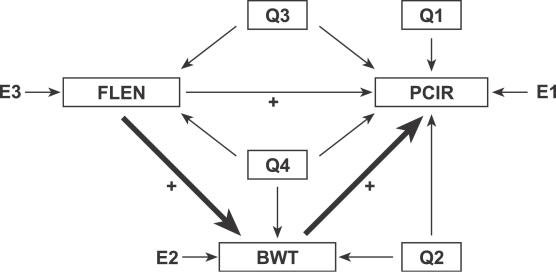
Structural Equation Model for Bone Geometry Genetic effects have been grouped. Sign and magnitude of path coefficients can be found in Table 5. Group Q1 includes loci with effects that are specific to PCIR (Q4@66, Q5@84, Q6@32, Q7@50, and Q11@68). Group Q2 includes loci have pleiotropic effects on PCIR and BWT (Q19@50, Q1@20, and Q8@0). Group Q3 includes loci with pleiotropic effects on PCIR and FLEN (Q10@64, Q5@52, Q15@10, and Q12@56). Group Q4 loci are pleiotropic loci that affect all three traits (Q12@2, Q2@66, and Q3@30). E1, E2, and E3 denote *N*(0,1) residual error.

**Figure 8:**
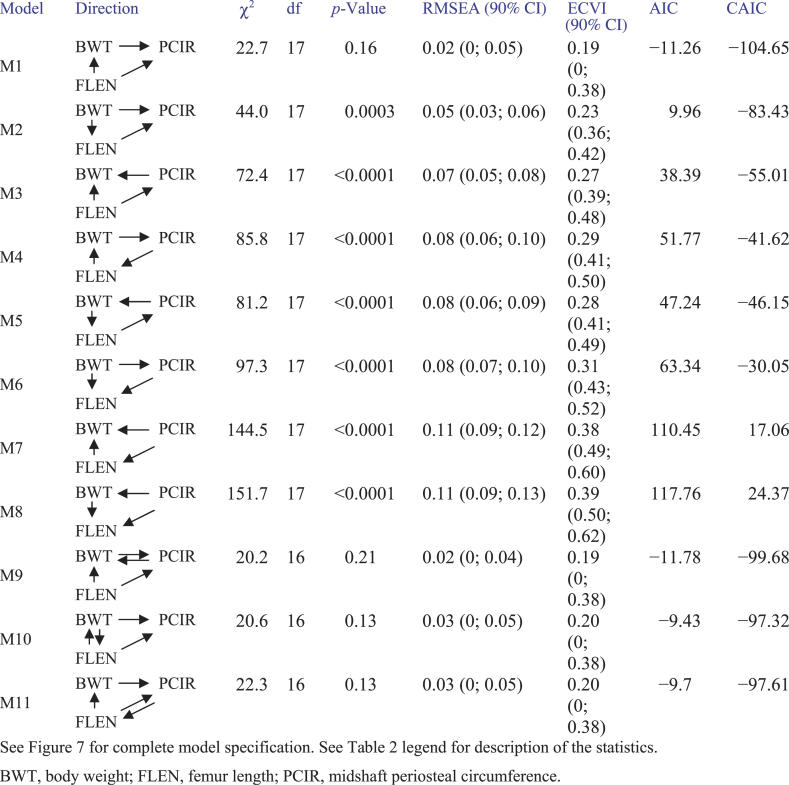
Model Comparison for the Bone Geometry Data Model comparisons for the bone geometry data were derived from the model in Figure 7 by varying the relationships among body weight, femur length, and periostial circumference.

Body weight and bone geometry phenotypes are known to be associated but the causal relationships among them are largely unknown [35]. Here we look at bone geometry data from a NZB × RF intercross population [23] and consider the relationships among femur length, midshaft PCIR, and body weight. QTLs associated with these traits have been described previously [23]. We employed an additive genetic model in the SEM because all QTL effects showed intralocus additivity. We developed an initial SEM (Figure 7 and Table 5) following the steps of model formulation, assessment, and refinement described in Materials and Methods. In order to resolve the causal relationships among the three phenotypes, we examined the complete set of 11 models listed in Figure 8. Models M1, M9, M10, and M11 each provide a close fit to the data, but t-tests for the extra path coefficients in models M9, M10, and M11 that are not found in model M1 are all nonsignificant (*t* = 1.68, −0.43, and 0.65, respectively). In addition, M1 has the smallest CAIC value relative to other models. We conclude that model M1 provides the best description of these data.

**Table 5:**
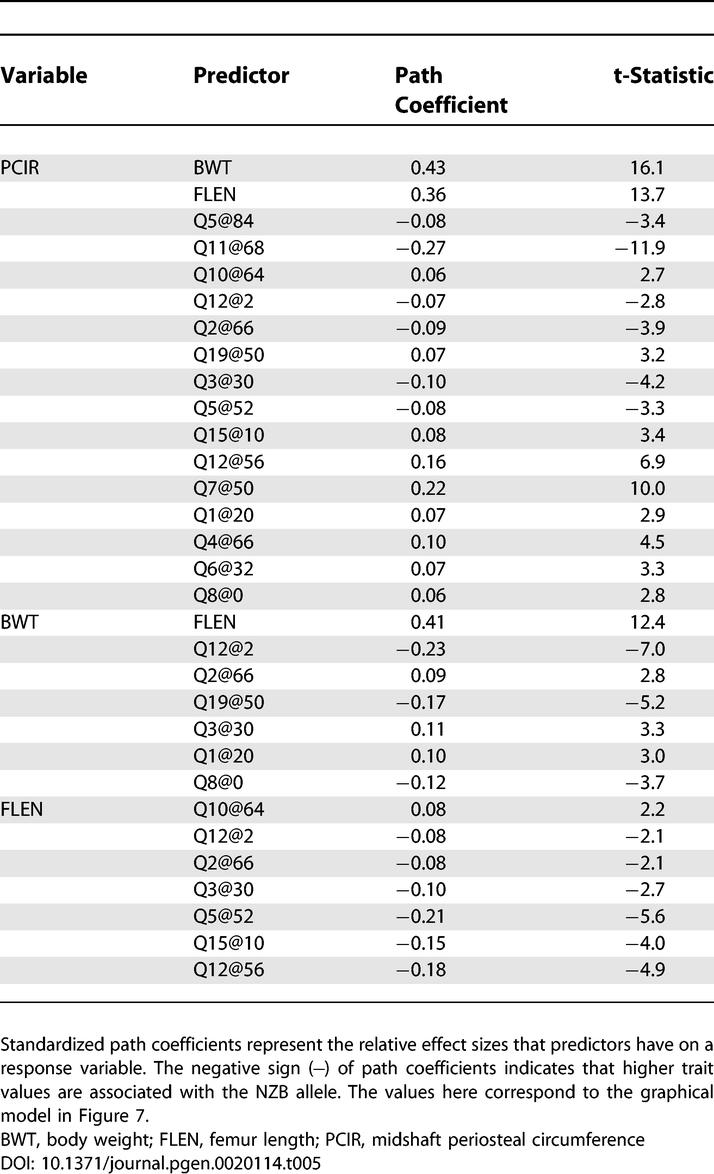
Structural Equations for the Bone Geometry Model

The graphical SEM is shown in Figure 7. Path coefficients and t-statistics are summarized in Table 5. The model explains 67.3% of the variance in PCIR, 29.5% of the variance in body weight, and 11.9% of the variance in femur length. The QTLs in group Q1 are specific to PCIR. The contributions are balanced in that Q5@84 and Q11@68 contribute high alleles from NZB, and Q7@50 and Q4@66 contribute low alleles from NZB. The QTLs in group Q2 affect both PCIR and body weight. Path coefficients indicate that Q12@2 and Q19@50 are primarily body weight QTLs. Other members of Q2 (Q2@66, Q3@30, and Q8@0) show opposing effects on body weight and PCIR. Causal connections among the traits are consistent with our prior expectations for this study. Muscle mass, a major determinant of body weight, is associated with the length of the femur, and a mouse with greater body weight will tend to have thicker bones. Again, we should use caution in these interpretations as there is certainly some cross-talk among these traits as well as unobserved factors that could influence the relationships among the measured variables. The SEM describes the net effects of many factors.

## Discussion

In experimental crosses, meiosis serves as a randomization mechanism that distributes naturally occurring genetic variation in a combinatorial fashion among a set of cross progeny. Genetically randomized populations share the properties of statistically designed experiments that provide a basis for causal inference. This is consistent with the notion that causation flows from genes to phenotypes. We propose that the inference of causal direction can be extended to include relationships among phenotypes and demonstrate that causal hypotheses can be tested. When models are nested, likelihood ratio tests can be applied. For non-nested sets of models, penalized likelihood methods such as AIC or CAIC can be used to identify good models. However, the credibility of any causal hypothesis must be judged by biological standards and not solely on statistical evidence. From this perspective, we view SEM as a device for generating causal hypotheses to be tested by subsequent experimentation.

As descriptive models, SEMs are useful for identifying the nature of pleiotropic QTL effects. The phenotypes that we choose to measure and the QTLs that we detect will rarely, if ever, occur in one-to-one correspondence. Allelic variation at a locus will often impact multiple traits, and these effects may be mediated through multiple physiological pathways. SEMs provide a quantitative description of the entire system of QTLs and phenotypes. In our analysis of the SM × NZB fat pad data we identified distinct classes of QTLs that affect body size and adiposity. The SEM analysis reveals a shortcoming of standardized variables, such body mass index and adiposity index, that are widely used in obesity research. This suggests that a reexamination of obesity QTLs in the literature may be worthwhile. The SEM is able to distinguish QTLs that affect adiposity from those that affect body size, whereas standardized measures could confound these two distinct types of effects.

Specification of a good initial model is perhaps the most important step in structural equation modeling of genetic data. We have developed a strategy to specify the initial model based on genome-wide QTL scans with conditioning on intermediate phenotypes. For the data examined here, these initial models fit reasonably well and final models were obtained with minor or no refinements. Our model development strategy incorporates elements of both exploratory and confirmatory SEM, because the initial model structure has been shaped by considering the same data that are used to assess and refine the SEM. Goodness-of-fit criteria are most informative regarding a lack of fit, and are subject to the error of overfitting. In the models presented here, most of the residual df derive from the known structure of linkage among QTLs. We used additive genetic models, but also described approaches to include dominance and epistasis (Figure S2). Although these are important features of realistic genetic models, they may artificially inflate the df and further degrade the utility of an overall goodness of fit statistic. Therefore, model selection statistics and likelihood ratio tests are the most appropriate methods for assessing the structure of the phenotype component of the model [30].

Sample size is an important consideration for both QTL mapping and for structural modeling. We recommend using at least 200 mice in an intercross mapping design to ensure that all large to moderate effect QTL are detected. Structural modeling relies on estimated variances and covariances and sample size will determine the precision of these estimates. Guidelines developed for SEM suggest that the sample size should be at least five, and preferably ten times the number of variables in the model [4]. Realistic sample sizes can impose tight limitations on the number of variables that can be studied simultaneously using SEM methods. We have applied SEM methods to small numbers of phenotypes and thus we were able to consider all possible structures for the trait component of the SEM. This may not be possible when larger sets of phenotypes are considered. Extensions of this method to high dimensional data, such as gene expression microarrays, may present additional challenges. However, the same principles should apply, which suggests that applications of causal inference in high dimensional data [36] deserve careful consideration.

Awareness of the limitations of any model is essential to its proper interpretation. Genetic mapping in line crosses provides only coarse resolution, and any apparently singular QTL may represent the composite effects of several tightly linked polymorphic loci. Tight linkage may be misinterpreted as pleiotropy. Important QTLs or epistatic interactions may not be detected and critical physiological parameters may not have been measured. When important components of a system are missing, correlations or spurious effects may be induced that would otherwise not be inferred. For example, significant covariances among residual errors for all of the fat pad traits suggest that nongenetic factors may be involved. Food intake is a plausible source of the correlated variation. Latent variables are useful when multiple traits are highly correlated and are presumed to share a common causal factor that is not directly measured, or one that is not measurable.

Structural equation modeling provides a powerful descriptive approach to the genetic analysis of multiple traits. SEMs allow characterization of pleiotropic and heterogeneous genetic effects of multiple loci on multiple traits as well as the physiological interactions among traits. With both graphical and algebraic representations, SEMs provide an intuitive and precise description of the genetic architecture of a complex system.

## Supporting Information

Figure S1 Genome Scans for Each of the Fat Pad Weights Genome-wide scans for inguinal (A), gonadal (B), peritoneal (C), and mesenteric (D) fat pad traits, based on a single QTL model. Through (A) to (D), the top scan is unconditioned for lean body weight; the middle scan is conditioned for lean body weight; and the bottom scan is the difference in LOD scores between conditioning and unconditioning. ING, inguinal fat pad weight; GON, gonadal fat pad weight; LBWT, lean body weight; MES, mesenteric fat pad weight and PERI, peritoneal fat pad weight. Phenotypes are square root transformed.
(585 KB PDF)

Figure S2 Structural Equation Models for Individual Fat Pad traits Structural equation models for inguinal (A), gonadal (B), peritoneal (C), and mesenteric (D) fat pad traits. See Figure 4 legend for details. For QTL-dominant effect, a positive sign (+) is assigned when the effect of heterozygous genotype is not different from that of homozygous SM allele relative to homozygous NZB allele. Genetic loci are parameterized using the Cockerham model.
(521 KB PDF)

Table S1 Path Analysis of the Inguinal Fat Pad Model (29 KB DOC)

Table S2 Path Analysis of the Gonadal Fat Pad Model (28 KB DOC)

Table S3 Path Analysis of the Peritoneal Fat Pad Model (28 KB DOC)

Table S4 Path Analysis of the Mesenteric Fat Pad Model (29 KB DOC)

## Abbreviations

A: additive
D: dominant
AIC: Akaike's information criterion
df: degrees of freedom
ECVI: expected value of the cross-validation index
LOD: log of odds ratio
PCIR: periosteal circumference
QTL: quantitative trait locus
SEM: structural equation model
