# Supplementary material for: Structural Model Analysis of Multiple Quantitative Traits
Source: PLoS Genet. 2006 Jul 21;2(7):e114. doi: 10.1371/journal.pgen.0020114 (PMC1513264; doi:10.1371/journal.pgen.0020114)

Supplemental Figure 1

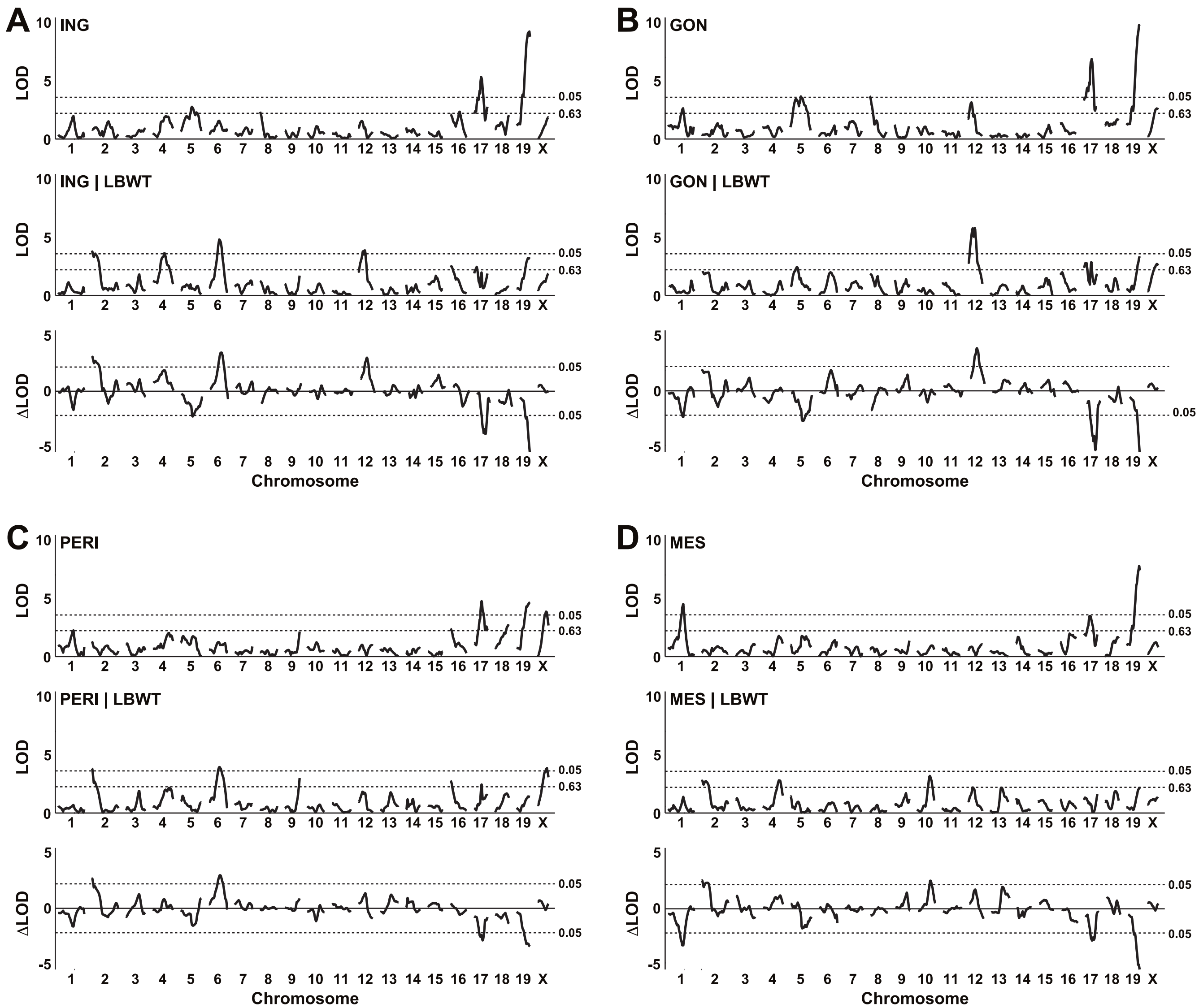

Supplement: Supplementary file 1 [file pgen.0020114.sg001.pdf]
