# Supplementary material for: Structural Model Analysis of Multiple Quantitative Traits
Source: PLoS Genet. 2006 Jul 21;2(7):e114. doi: 10.1371/journal.pgen.0020114 (PMC1513264; doi:10.1371/journal.pgen.0020114)
Supplement: Supplementary file 3 [file pgen.0020114.st001.doc]

Path	Effect	Pcorr

[Q19@](mailto:Q12@0)52A	ING (-)	-0.1157	-0.1564

[Q19@](mailto:Q12@0)52A	**LBWT**	ING (-,+)	-0.0966

[Q19@](mailto:Q12@0)52D	**LBWT**	ING (-, +)	-0.0691

# Total -0.2824

[Q17@](mailto:Q12@0)28A	ING (-)	-0.0902	-0.1343

[Q17@](mailto:Q17@)28A	**LBWT**	ING (-,+)	-0.0807

# Total -0.1709

[Q2@](mailto:Q12@0)2A	ING (+)	0.1301	0.1629

[Q2@](mailto:Q17@)2A	**LBWT**	ING (-,+)	-0.0669

# Total 0.0632

[Q6@](mailto:Q12@0)46A	ING (+)	0.1338	0.1695

[Q6@](mailto:Q17@)46A	**LBWT**	ING (-,+)	-0.0829

# Total 0.0509
