# Supplementary material for: Structural Model Analysis of Multiple Quantitative Traits
Source: PLoS Genet. 2006 Jul 21;2(7):e114. doi: 10.1371/journal.pgen.0020114 (PMC1513264; doi:10.1371/journal.pgen.0020114)
Supplement: Supplementary file 4 [file pgen.0020114.st002.doc]

Path	Effect	Pcorr

[Q19@](mailto:Q12@0)52A	GON (-)	-0.1021	-0.1241

[Q19@](mailto:Q12@0)52A	**LBWT**	GON (-,+)	-0.0886

[Q19@](mailto:Q12@0)52D	**LBWT**	GON (-,+)	-0.0627

# Total -0.2534

[Q17@](mailto:Q12@0)28A	GON (-)	-0.1229	-0.1572

[Q17@](mailto:Q17@)28A	**LBWT**	GON (-,+)	-0.0873

# Total -0.2102

[Q1@](mailto:Q12@0)62A	GON (-)	-0.0747	-0.1050

[Q1@](mailto:Q17@)62A	**LBWT**	GON (-,+)	-0.0867

# Total -0.1614

[Q8@](mailto:Q12@0)0A	GON (-)	-0.1038	-0.1332

[Q8@](mailto:Q17@)0A	**LBWT**	GON (-,+)	-0.0552

# Total -0.1590
