# Supplementary material for: Structural Model Analysis of Multiple Quantitative Traits
Source: PLoS Genet. 2006 Jul 21;2(7):e114. doi: 10.1371/journal.pgen.0020114 (PMC1513264; doi:10.1371/journal.pgen.0020114)
Supplement: Supplementary file 5 [file pgen.0020114.st003.doc]

Path	Net Effect	Pcorr

[Q19@](mailto:Q12@0)52A	PERI (-)	-0.0821	-0.1035

[Q19@](mailto:Q12@0)52A	**LBWT**	PERI (-,+)	-0.0850

[Q19@](mailto:Q12@0)52D	**LBWT**	PERI (-,+)	-0.0494

# Total -0.2165

[Q17@](mailto:Q12@0)28A	PERI (-)	-0.0865	-0.1004

[Q17@](mailto:Q17@)28A	**LBWT**	PERI (-,+)	-0.0848

# Total -0.1713

[Q2@](mailto:Q12@0)2A	PERI (+)	0.1500	0.1751

[Q2@](mailto:Q17@)2A	**LBWT**	PERI (-,+)	-0.0517

# Total 0.0983

[Q6@](mailto:Q12@0)46A	PERI (+)	0.1469	0.1828

[Q6@](mailto:Q17@)46A	**LBWT**	PERI (-,+)	-0.0597

# Total 0.0872
