# Supplementary material for: Structural Model Analysis of Multiple Quantitative Traits
Source: PLoS Genet. 2006 Jul 21;2(7):e114. doi: 10.1371/journal.pgen.0020114 (PMC1513264; doi:10.1371/journal.pgen.0020114)
Supplement: Supplementary file 6 [file pgen.0020114.st004.doc]

Path	Effect	Pcorr

[Q19@](mailto:Q12@0)52A	MES (-)	-0.0973	-0.1492

[Q19@](mailto:Q12@0)52A	**LBWT**	MES (-,+)	-0.1035

[Q19@](mailto:Q12@0)52D	**LBWT**	MES (-,+)	-0.0710

# Total -0.2718

[Q17@](mailto:Q12@0)28A	MES (-)	-0.0685	-0.0946

[Q17@](mailto:Q17@)28A	**LBWT**	MES (-,+)	-0.0971

# Total -0.1656

[Q1@](mailto:Q1@)62A	MES (-)	-0.0901	-0.1218

[Q1@](mailto:Q1@)62A	**LBWT**	MES (-,+)	-0.0917

# Total -0.1818

[Q2@](mailto:Q12@0)2A	MES (+)	0.1240	0.1675

[Q2@](mailto:Q17@)2A	**LBWT**	MES (-,+)	-0.0631

# Total 0.0609

[Q10@](mailto:Q12@0)52A	MES (+)	0.1258	0.1833

[Q10@](mailto:Q17@)52A	**LBWT**	MES (-,+)	-0.0511

# Total 0.0747
